# Argatroban therapy for heparin-induced thrombocytopenia in ICU patients with multiple organ dysfunction syndrome: a retrospective study

**DOI:** 10.1186/cc9024

**Published:** 2010-05-20

**Authors:** Bernd Saugel, Veit Phillip, Georg Moessmer, Roland M Schmid, Wolfgang Huber

**Affiliations:** 1 II. Medizinische Klinik, Klinikum rechts der Isar der Technischen Universität München, Ismaningerstr. 22, 81675 München, Germany; 2 Institut für klinische Chemie und Pathobiochemie, Klinikum rechts der Isar der Technischen Universität München, Ismaningerstr. 22, 81675 München, Germany

## Abstract

**Introduction:**

Heparin-induced thrombocytopenia (HIT) is a serious, prothrombotic, immune-mediated adverse reaction triggered by heparin therapy. When HIT is diagnosed or suspected, heparins should be discontinued, and an alternative, fast-acting, parenteral, nonheparin anticoagulation such as argatroban should be initiated. Limited and inconsistent data exist about dosing of argatroban in intensive care unit (ICU) patients with critical illnesses.

**Methods:**

Retrospective analysis of 12 ICU patients with multiple organ dysfunction syndrome (MODS) treated with argatroban for suspected or diagnosed HIT.

**Results:**

The 12 ICU patients with a mean platelet count of 46,000 ± 30,310 had a mean APACHE II score of 26.7 ± 7.8 on ICU admission and a mean SAPS II score of 61.5 ± 16.3 on the first day of argatroban administration. A mean argatroban starting dose of 0.32 ± 0.25 μg/kg/min (min, 0.04; max, 0.83) was used to achieve activated partial thromboplastin times (aPTTs) >60 sec or aPTTs of 1.5 to 3 times the baseline aPTT. Adjustment to aPTT required dose reduction in six (50%) patients. Patients were treated for a mean of 5.5 ± 3.3 days. The final mean dose in these critically ill patients was 0.24 ± 0.16 μg/kg/min, which is about one eighth of the usually recommended dose and even markedly lower than the previously suggested dose for critically ill ICU patients. In all patients, desired levels of anticoagulation were achieved. The mean argatroban dose was significantly lower in patients with hepatic insufficiency compared with patients without hepatic impairment (0.10 ± 0.06 μg/kg/min versus 0.31 ± 0.14 μg/kg/min; *P *= 0.026). The mean argatroban dose was significantly correlated with serum bilirubin (*r *= -0.739; *P *= 0.006).

**Conclusions:**

ICU Patients with MODS and HIT can be effectively treated with argatroban. A decrease in the initial dosage is mandatory in this patient population. Further studies are needed to investigate argatroban elimination and dosage adjustments for critically ill patients.

## Introduction

Heparin-induced thrombocytopenia (HIT) is a serious, prothrombotic, immune-mediated adverse reaction triggered by heparin therapy [1]. HIT is more often caused by unfractionated heparin than by low-molecular-weight heparin [2]. In HIT, antibodies of immunoglobulin G class bind to a complex of heparin and platelet factor 4, resulting in platelet activation and excessive thrombin generation, leading to thrombocytopenia, a hypercoagulable state, and often to thrombosis. Unless alternative anticoagulation is initiated, the risk of arterial or venous thromboembolic complications in HIT is about 30% to 75% of cases, leading to limb amputations in 10% to 20% and to death in 20% to 30% of cases [3-6]. If platelet count decreases to ≥50% or thrombosis occurs between day 5 and 14 of heparin therapy, or both, HIT should be suspected [7]. In patients with recent heparin exposure within the previous 100 days, clinically significant HIT antibodies may still circulate and can therefore cause an abrupt decrease in platelet count with restarting of heparin treatment [8].

For laboratory diagnosis of HIT antibodies, antigen assays as well as functional assays (platelet activation) are used, both showing a high sensitivity [7,9].

According to consensus guidelines, when HIT, with or without thrombosis, is diagnosed or strongly suspected, heparins should be immediately discontinued and an alternative, fast-acting, parenteral, nonheparin anticoagulation should be promptly initiated [7]. Three alternative parenteral anticoagulants have been approved for use in HIT: the heparinoid danaparoid and the direct thrombin inhibitors, lepirudin and argatroban.

Argatroban is a synthetic direct thrombin inhibitor, derived from L-arginine, that selectively and reversibly inhibits free and clot-bound thrombin at the catalytic site [10]. Argatroban is predominantly hepatically metabolized [11]. Renal elimination of argatroban is minimal, and pharmacokinetic and pharmacodynamic parameters of argatroban have been demonstrated to be comparable between healthy subjects and non-HIT patients with different degrees of renal insufficiency [11-15]. In addition, argatroban anticoagulation has been used successfully during renal-replacement therapy in patients with and without HIT [15,16]. However, recent limited data suggested the consideration of kidney function before initiation of argatroban therapy in HIT [13,17,18].

The recommendation for initial dosing of argatroban in HIT is 2 μg/kg/min, adjusted as needed to achieve activated partial thromboplastin times (aPTTs) of 1.5 to 3 times the patient's baseline aPTT [5,19]. To account for the reduction in clearance, the recommended initial dose for patients with hepatic impairment is 0.5 μg/kg/min.

Despite the availability of these recommendations, limited and inconsistent data exist about dosing patterns, efficacy, and safety of argatroban therapy in intensive care unit (ICU) patients with critical illness or multiple organ dysfunction syndrome (MODS). Studies on argatroban therapy in critically ill patients with MODS and suspected or diagnosed HIT are limited to very small case series with conflicting results [13,14]. Previous data showed that no argatroban dose adjustment is needed in acutely ill patients [20]. In contrast, recent data indicated that the approved dosing regimens of the direct thrombin inhibitors are too high in critically ill ICU patients, especially with MODS [14,17,21]. A commonly suggested starting dose for ICU patients is 0.5 to 1.0 μg/kg/min, with adjustment according to aPTT (target range, 1.5 to 3 times or ≥60 sec) [13,21,22]. Further investigations are needed to ensure safe, appropriate dosing guidelines for the use of argatroban in the setting of critically ill ICU patients with HIT.

In our retrospective analysis, we evaluated critically ill ICU patients with MODS treated with argatroban for diagnosed or suspected HIT. The primary objective of this observational analysis was to demonstrate dosing-adjustment difficulties of argatroban, especially in the setting of MODS.

## Materials and methods

We retrospectively analyzed argatroban dosing patterns and anticoagulant responses in 12 consecutively selected adult patients with MODS who received argatroban for suspected or diagnosed HIT between March 2007 and March 2009 at the general ICU of a German university hospital (Klinikum rechts der Isar der Technischen Universität München, Munich, Germany). The patients were critically ill (defined as having an Acute Physiology and Chronic Health Evaluation II Score, APACHE II, higher than 15) and were treated for MODS involving two or more organ systems. The APACHE II score was calculated after admission of a patient to the ICU and, in addition, the Simplified Acute Physiology Score (SAPS II) was calculated on the first day of argatroban administration.

The general policy in our ICU is to stop all sources of heparin and initiate an alternative anticoagulant on reasonable suspicion of HIT. The choice of alternative anticoagulant agent and initial dose is at the discretion of the treating physician. The dose is generally adjusted to achieve aPTTs >60 sec or aPTTs of 1.5 to 3 times the baseline aPTT. HIT was defined as a decrease in platelet count to >150 × 10^9^/L or by >50%, starting at least 5 days after initiation of heparin exposure, provided that a more likely cause for the platelet decline has been ruled out. The aPTT was measured about 2 h after initial argatroban administration, and dose adjustments were made to maintain desired aPTT levels. The aPTT was assessed daily and 4 h after any dose adjustment. Data extracted from each patient chart included the demographics, previous heparin exposure, organ-failure status, heparin-induced platelet-activation (HIPA) test results (functional assay, platelet activation), each argatroban dose, as well as aPTT and International Normalized Ratio (INR) values.

A seriously reduced level of consciousness, Glasgow coma scale <12 (without head injury) or Cook and Palma score <12 was defined as cerebral involvement in MODS. Respiratory insufficiency was defined as necessity for noninvasive ventilation or mechanical ventilation. Need for administration of inotropic substances or vasopressors was documented as circulatory failure. A patient was considered to have hepatic insufficiency if the serum aspartate aminotransferase or alanine aminotransferase levels thrice exceeded the upper limit of normal. A patient was considered to have renal insufficiency if the creatinine clearance was <60 mL/min or the serum creatinine was >3.0 mg/dL, or both, or renal replacement therapy was needed.

Descriptive statistical analyses were performed by using Tinn-R statistical software. Results, where applicable, are reported as mean ± SD. To evaluate factors associated with the individual mean argatroban dose, we performed univariate analysis (Spearman correlation), including serum bilirubin, aspartate aminotransferase, Model of End-Stage Liver Disease (MELD) score, APACHE II score, and serum creatinine. The Wilcoxon test for unpaired measurements was applied to compare the mean argatroban dose in patients with or without hepatic or renal failure, respectively. Statistical significance was defined as a *P *value of < 0.05. Factors significantly correlated to the mean argatroban dose were included in a multiple regression analysis (backward selection) regarding the individual mean argatroban dose in a second step. In addition to the factors derived from Spearman correlation, a limited number of factors with high *a priori *probability of impact on the mean argatroban dose (such as APACHE II score) and markers of hepatic and renal failure were included in the multiple regression analysis. Statistical analysis was performed by using software (SPSS. version 16; SPSS inc., Chicago, IL, USA). The study was approved by the local ethics committee. The need for informed consent was waived for this retrospective analysis of data.

## Results

### Patients

Twelve (eight female and four male) critically ill ICU patients with a mean age of 70.0 ± 17.3 years and a mean weight of 69.5 ± 20.1 kg were enrolled in this study (Table 1). The mean APACHE II score on ICU admission and the mean SAPS II score on the day of initial argatroban administration were 26.7 ± 7.8 and 61.5 ± 16.3, respectively. All patients were treated for MODS with an involvement of two or more organ systems (Table 2), and eight (67%) patients were classified as having sepsis. Mechanical ventilation was needed in 10 (83%) patients, and administration of inotropic substances or vasopressors was necessary in seven (58%) patients. Renal insufficiency was observed in seven (58%) patients, and hepatic insufficiency in four (33%) patients. Five (42%) patients died during their ICU stay.

**Table 1:** Characteristics of patients, demographic parameters

Patient number	Sex	APACHE-II score	SAPS-II score	Main diagnosis	ICU survival	Cause of death
1	m	17	42	Cirrhosis of the liver	-	Pneumonia, sepsis
2	f	27	69	Pneumonia, sepsis	+	
3	f	31	78	Erysipelas, sepsis	+	
4	f	27	56	Liver failure	-	Pneumonia, sepsis
5	m	22	62	Intracerebral hemorrhage	+	
6	f	33	73	Pneumonia, sepsis	-	Peritonitis, sepsis
7	m	34	77	Pneumonia, sepsis	+	
8	f	17	44	Pneumonia, sepsis	+	
9	f	34	79	Pneumonia, sepsis	-	Pneumonia, sepsis
10	f	40	74	Pulmonary embolism	-	Heart insufficiency
11	f	19	54	Retroperitoneal hemorrhage	+	
12	m	19	30	Cirrhosis of the liver	+	
	**8 f/4 m**	**26.7 ± 7.8**	**61.5 ± 16.3**		**7+/5-**	

Showing sex (m, male; f, female), APACHE II score on ICU admission, SAPS-II score on the day of first argatroban administration, main diagnosis on ICU admission, ICU survival (+, survived; -, died), and cause of death. Data are provided for each patient as mean ± SD where possible. APACHE II, Acute Physiology and Chronic Health Evaluation II Score; ICU, intensive care unit; SAPS II, Simplified Acute Physiology Score.

**Table 2:** Characteristics of patients and organ dysfunction

Patient number	CNS involvement	Respiratory insufficiency	Circulatory failure	Renal insufficiency	Hepatic insufficiency	Sepsis
1	**+**	**-**	**-**	**+**	**+**	**+**
2	**+**	**+**	**-**	**-**	**-**	**+**
3	**+**	**+**	**+**	**+**	**-**	**+**
4	**+**	**+**	**+**	**-**	**+**	**+**
5	**+**	**+**	**-**	**-**	**-**	**-**
6	**+**	**+**	**+**	**+**	**-**	**+**
7	**+**	**+**	**-**	**+**	**-**	**+**
8	**+**	**+**	**+**	**-**	**-**	**-**
9	**+**	**+**	**+**	**+**	**+**	**+**
10	**+**	**+**	**+**	**+**	**-**	**+**
11	**+**	**+**	**-**	**-**	**-**	**-**
12	**+**	**-**	**-**	**+**	**+**	**-**
**Total**	12	10	6	7	4	8

Showing organ dysfunction and diagnosis of sepsis at the beginning of argatroban therapy. Data are provided for each patient.

### Argatroban anticoagulation

All patients were treated with argatroban anticoagulation for suspected HIT. When argatroban therapy was started, mean thrombocyte count was 46,000 ± 30,310/μl (min, 9,000; max, 93,000) (Table 3). In six (50%) patients, suspicion of HIT was confirmed by laboratory tests (functional assay, HIPA test). Argatroban anticoagulation in this study was started at a low dose, and no loading dose of argatroban was used: The mean argatroban starting dose was 0.32 ± 0.25 μg/kg/min (min, 0.04 μg/kg/min; max, 0.83 μg/kg/min) to achieve aPTTs >60 sec or aPTTs of 1.5 to 3 times the baseline aPTT. Desired levels of anticoagulation were achieved in all patients. In the critically ill patients in this study, the aPTT was elevated at baseline (median value of 49 ± 13 sec) and increased further (median of 66 ± 18 sec) by the first assessment after initiating argatroban. In accordance to that, baseline INR values increased from 1.23 ± 0.38 to 1.49 ± 0.23 after starting argatroban. Despite the very low starting dose, adjustment to aPTT required dose reduction in six (50%; one patient with renal and hepatic failure, two patients with renal insufficiency, one patient with hepatic impairment, two patients with neither hepatic nor renal failure). Patients were treated for a mean of 5.5 ± 3.3 days (min, 1 day; max, 11 days). The final mean dose in these critically ill ICU patients was 0.24 ± 0.16 μg/kg/min (min, 0.02 μg/kg/min; max, 0.48 μg/kg/min).

**Table 3:** Argatroban therapy

Patient	HIPA test	Platelet count (×1,000/μL)	INR before A	INR after A	aPTT before A (sec)	aPTT after A (sec)	aPTT mean during A therapy (sec)	A starting dose (μg/kg/min)	A mean dose (μg/kg/min)
1	**+**	93	1.2	1.6	44	80	68.4	0.08	0.02
2	**+**	48	0.8	1.2	39	62	56.3	0.48	0.48
3	**-**	18	1.1	1.6	63	106	88.7	0.56	0.37
4	**+**	9	2.1	1.5	79	57	93.2	0.22	0.15
5	**+**	38	1.1	1.6	29	51	36.4	0.04	0.14
6	**-**	31	1.0	1.0	48	51	60.5	0.14	0.14
7	**-**	87	1.1	1.4	46	53	62.5	0.46	0.46
8	**-**	46	1.2	1.6	52	87	61.3	0.83	0.30
9	**-**	19	1.9	1.9	54	63	63.0	0.08	0.08
10	**-**	15	1.1	1.6	58	75	67.5	0.56	0.42
11	**+**	90	1.0	1.5	42	56	68.5	0.22	0.20
12	**+**	58	1.2	1.4	36	47	51.5	0.14	0.14
	**6+/6-**	**46 ± 30**	**1.23 ± 0.38**	**1.49 ± 0.23**	**49 ± 13**	**66 ± 18**	**64.8 ± 15.1**	**0.32 ± 0.25**	**0.24 ± 0.16**

For each patient (pt), HIPA-test results (+, positive, - = negative), platelet count (×1,000 per microliter), INR/aPTT values before argatroban therapy, and INR/aPTT values at first assessment after starting argatroban are provided. Mean aPTTs during argatroban therapy, argatroban starting doses, and argatroban mean doses are shown. Data are provided for each patient and as mean ± SD where possible. A, argatroban; aPTT, activated partial thromboplastin time; HIPA, heparin-induced platelet activation; INR, International Normalized Ratio.

The mean argatroban dose was significantly different in patients with hepatic insufficiency compared with patients without hepatic impairment (0.10 ± 0.06 μg/kg/min versus 0.31 ± 0.14 μg/kg/min; *P *= 0.026). In contrast, no difference was found in mean argatroban dose in patients with or without renal insufficiency (0.23 ± 0.18 μg/kg/min versus 0.25 ± 0.14 μg/kg/min; *P *= 0.530).

Univariate analysis demonstrated that the mean argatroban dose was significantly correlated with serum bilirubin (*r *= -0.739; *P *= 0.006) but not with aspartate aminotransferase (*r *= -0.321; *P *= 0.309), MELD score (*r *= -0.400; *P *= 0.600), APACHE II score (*r *= 0.330; *P *= 0.295) or serum creatinine (*r *= -0.198; *P *= 0.538). Subsequently we performed multiple regression analysis regarding mean argatroban dose, showing that among all analyzed variables (APACHE II, serum creatinine, presence of hepatic insufficiency, presence of renal insufficiency), only the presence of hepatic insufficiency was independently associated with the mean argatroban dose (*r *= 0.676; *P *= 0.016).

No bleeding complications or other adverse events occurred in the patient population of this study during anticoagulation therapy with argatroban. Furthermore, no arterial or venous thromboembolic complications appeared in the 12 patients treated with argatroban.

## Discussion

In critically ill ICU patients, the recognition, diagnosis, and therapy of HIT is very difficult. Thrombocytopenia (mostly due to sepsis or hemodilution) is a very common laboratory finding, occurring in ~30% to 50% of patients in the medical ICU [23]. However, the diagnosis of HIT should be based on clinical considerations and treatment should not be delayed, pending laboratory confirmation [3,7]. On suspicion of HIT, all sources of heparin should be eliminated and an alternative anticoagulant must be initiated [7].

Three alternative parenteral anticoagulants have been approved for treatment of HIT: the heparinoid danaparoid and the direct thrombin inhibitors lepirudin and argatroban [5,24-26].

In the special setting of critically ill ICU patients, argatroban has some advantages over lepirudin [14]. Lepirudin is renally cleared and associated with an increased elimination half-life and bleeding risk in renal failure [27]. Argatroban is metabolized hepatically and eliminated in the feces through biliary excretion [11]. Many studies indicated that renal insufficiency does not influence pharmacokinetic or pharmacodynamic parameters of argatroban and that argatroban is well tolerated and provides adequate anticoagulation in patients with renal dysfunction or failure as well as during renal replacement therapy [11-14,16,28-30]. In contrast, recent limited data suggested consideration of kidney function and dose adjustment in HIT therapy with argatroban [13,16-18]. Moreover, the elimination half-life of argatroban (about 39 to 51 min in healthy subjects) is reduced by 50% in comparison to lepirudin [11].

Two prospective, multicenter, historical controlled studies and reanalyses of their combined data demonstrated that the use of argatroban resulted in reducing the composite end point of death, amputation, or new thrombosis in HIT patients, with particular benefit in decreasing new thrombosis without increasing bleeding [5,19].

The recommended initial dose of argatroban for the prophylaxis or treatment of thrombosis in HIT is 2 μg/kg/min (0.5 μg/kg/min for patients with hepatic impairment) with following adjustment to aPTTs of 1.5 to 3 times the baseline aPTT [5,19].

Very limited and inconsistent data exist about dosing patterns of argatroban therapy in ICU patients with critical illness [13,14]. Some data demonstrate that no dose adjustment is required in argatroban therapy of acutely ill patients [20]. Other data indicate that the pharmacokinetics and clearance of argatroban seem to be substantially altered in critically ill patients [13,14,17,21,31,32]. Especially in the setting of sepsis and MODS, hepatic clearance of argatroban may be significantly reduced. Hepatic metabolism in these patients may be influenced by reduced cardiac output, circulatory distributory failure, and/or disseminated intravascular coagulation, resulting in decreased hepatic perfusion. Therefore, some authors suggest an argatroban starting dose for ICU patients of 0.5 to 1.0 μg/kg/min [13,21,22].

To gain additional knowledge about dosing-adjustment problems in the use of argatroban in the setting of critical illness and HIT, we retrospectively evaluated 12 ICU patients with MODS treated with argatroban. All patients had developed thrombocytopenia after heparin exposure and had argatroban treatment initiated for suspected HIT whether the diagnosis was ultimately confirmed. The patients in this series were critically ill at the time of argatroban initiation (APACHE II score, 26.7 ± 7.8; SAPS II score, 61.5 ± 16.3) and were treated for MODS. All patients had a platelet count <100,000/μl (mean platelet count at the time of starting argatroban anticoagulation, 46,000 ± 30,310/μl). The overall clinical status of our patient population was probably more critical compared with those reported previously. Our patients were treated with a median argatroban starting dose of 0.32 ± 0.25 μg/kg/min. Further dose reduction was needed in 50% of the patients. The final required median argatroban dose was 0.24 ± 0.16 μg/kg/min, representing one eighth of the usually recommended dose. Desired levels of anticoagulation were promptly achieved in all patients.

Compared with previous data, the argatroban dose we applied in our MODS patients was markedly lower than the previously suggested dose for ICU patients, probably reflecting the degree of illness in our patients; likely a tendency existed toward targeting the lower end of the therapeutic aPTT range in these very ill patients.

For example, one recent study investigated argatroban treatment in critically ill ICU patients with HIT II and the necessity for continuous renal-replacement therapy [16]. In this study, Link *et al. *[16] developed recommendations for argatroban dosing during continuous renal-replacement therapy: They used an initial argatroban bolus of 100 μg/kg followed by continuous infusion of argatroban. In contrast, no bolus of argatroban was used in our study to avoid a peak response of anticoagulation with the risk of bleeding complications. The average rate of argatroban in the study of Link *et al. *was higher compared with our study (0.70 μg/kg/min versus 0.24 μg/kg/min). Although both studies investigate argatroban treatment in critically ill patients, our study population of patients with MODS is clearly different from the patients included in the study of Link *et al. *In contrast to our study, Link *et al. *included only patients receiving continuous renal-replacement therapy. Argatroban was applied into the extracorporeal circulation (prefilter injection/infusion). Furthermore, none of the critically ill patients included in the Link study had a previous history of liver disease. Mean SAPS-II score was lower in this study population compared with that in our study (45 versus 61.5 points).

In general, in critically ill patients with MODS therapeutic interventions are a special challenge. Multifactorial changes in drug disposition and effect occur in these patients, resulting from drug/patient, drug/disease, and drug/drug interactions [33]. In particular, the liver as the primary site of biotransformation can be influenced manifestly, and hepatic impairment is associated with decreased systemic clearance and increased elimination half-life of argatroban [11,22].

## Conclusions

The results of our study suggest that patients with MODS and HIT can be effectively treated by using argatroban anticoagulation. A high index of suspicion is required in diagnosing HIT in these complex patients. However, in critically ill patients with MODS, the dosing of argatroban has to be adjusted. These data do not support the current recommendation of 0.5 to 1.0 μg/kg/min in patients with critical illness as a reasonable, conservative initial dosage of argatroban. To avoid excessive anticoagulation and bleeding complications, argatroban should be initiated at a markedly reduced dose of about one tenth to one eighth of the recommended 2 μg/kg/min in ICU patients with MODS. Because achievement of steady-state anticoagulation will be delayed in this patient population, aPTT must be checked at close intervals after drug initiation or dose change to ensure that the desired level of anticoagulation is achieved. Further studies are needed to investigate argatroban elimination and dosage adjustments for ICU patients with MODS.

## Key messages

• Patients with MODS and HIT can be effectively treated by using argatroban anticoagulation.

• In critically ill patients with MODS, the dosing of argatroban has to be adjusted.

• To avoid excessive anticoagulation and bleeding complications, argatroban should be initiated at a markedly reduced dose.

## Abbreviations

APACHE II: Acute Physiology and Chronic Health Evaluation II Score; aPTT: activated partial thromboplastin time; HIPA: heparin-induced platelet activation; HIT: heparin-induced thrombocytopenia; ICU: intensive care unit; INR: International Normalized Ratio; MELD score: Model of End Stage Liver Disease score; MODS: multiple organ dysfunction syndrome; SAPS II: Simplified Acute Physiology Score.

## Competing interests

The authors declare that they have no competing interests.

## Authors' contributions

BS, VP, and GM contributed to the conception and design of the study. They were responsible for acquisition, analysis, and interpretation of data. BS drafted the manuscript. RMS and WH participated in its design and coordination and helped to draft the manuscript.
